# In vivo evaluation of nanostructured lipid carrier systems (NLCs) in mice bearing prostate cancer tumours

**DOI:** 10.1007/s13346-021-01095-1

**Published:** 2021-11-29

**Authors:** Mushfiq Akanda, Giulia Getti, Dennis Douroumis

**Affiliations:** 1 Medway School of Science, Faculty of Engineering and Science, University of Greenwich, Kent, ME4 4TB UK; 2 Centre for Innovation & Process Engineering Research, Chatam Maritime, Kent, ME4 4TB UK

**Keywords:** Nanostructured lipid carriers, Curcumin, Prostate cancer, In vivo, Apoptosis, Cellular uptake

## Abstract

Nanostructure lipid carriers (NLCs) were developed for the delivery of curmumin (CRN), a potent anticancer agent with low bioavailability, for the treatment of prostate cancer. NLCs prepared using high pressure homogenization (HPH) with around 150 nm particle size, − 40 V ζ-potential and excellent long-term stability. Cellular uptake of CRN-SLN showed nanoparticle localization in the cytoplasm around the nucleus. CRN-NLCs were assessed using flow cytometry and found to cause early and late apoptotic events at 100 μg/ml CRN concentrations. CRN-NLC nanoparticles were administrated to nude mice with LNCaP prostate cancer xenografts and demonstrated substantial tumour volume suppression (40%) with no weight loss compared to pure CRN (ethanolic solution). Overall, NLCs were proved a suitable carrier for passive drug delivery and cancer treatment.

**Graphical abstract:**

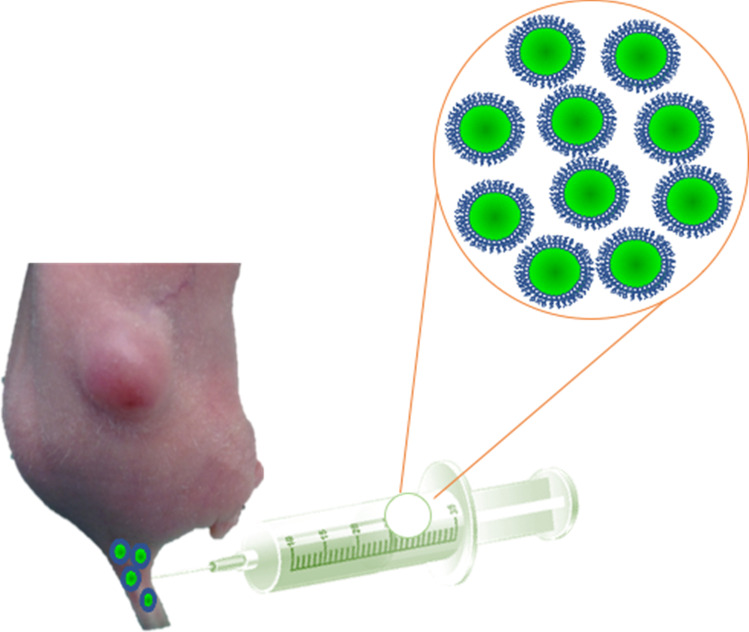

**Supplementary information:**

The online version contains supplementary material available at 10.1007/s13346-021-01095-1.

## Introduction

Nanostructured lipid carriers (NLCs) are versatile drug delivery systems that have been employed for the delivery of drug substances with enhanced clinical efficacy. Their wide applicability lies on the unique features they present such as increased drug encapsulations, long term physical and chemical stability of the encapsulated drug, surface functionalization and site-specific targeting [1, 2]. Furthermore, they can be easily produced using high pressure homogenization (HPH) under aseptic conditions while scale-up can be performed in a batch or continuous mode reaching a capacity of 100–150 kg/h.

NLCs were introduced by Rainer H. Müller (Berlin/Germany) and Maria Rosa Gasco (Turin/Italy) comprising solid and liquid lipid blends which result in imperfection of the lipid matrix due to large distances between the fatty acid chains [3]. Numerous studies have demonstrated why NLCs are considered to be a superior drug delivery system compared to other nano lipidic delivery systems. Alam et al. developed isradipine-loaded NLCs for the management of hypertension and myocardial infraction [4]. Clinical studies showed that NLC treatment prevents significantly the antioxidant status and ultrastructural changes of the heart due to the isradipine increased bioavailability. Thermo-reversible NLCs were used to form in situ implants and achieve sustained release of estradiol valearate [5]. The developed NLC formulations showed an increase of 16.8-fold in AUC and 40-fold of Cmax when compared to a commercial suspension.

NLCs have been also used as carriers for transdermal delivery due to the increase of solubility of water insoluble drug substances [4, 6–10]. A dermatokinetic assessment involved the administration of luliconazole-loaded NLCs with ultra-small particle size (< 100 nm) for antifungal activity [6]. Nanoparticles were loaded in a topical gel, and clinical results showed twofold increase of Cmax and threefold for the AUC compared to a marketed cream. Recently, a novel NLC formulation was designed for co-delivery of tacrolimus and siRNA for the treatment of psoriasis [11]. The in vivo studies confirmed the superiority of NLCs with significant reduction of the cytokine TNF-α expression and demonstrated the synergistic effect of tacrolimus and TNF-α siRNA with a sevenfold increase. In other studies, NLCs have been reported for brain targeting to treat Alzheimer’s disease [12] or for lymph targeting and the delivery of antiHIV agents [13].

However, NCLs have been extensively used for cancer treatment combined with chemotherapy to overcome challenges associated with tumours [14, 15]. In a recent study, surface-engineered NLCs were developed for combined chemotherapy through the delivery of docetaxel and curcumin by conjugating folic acid. The dual drug delivery offers several advantages such as dose reduction, overcoming of multi-drug resistance and can result in synergistic effects. The in vivo studies in murine lung carcinoma showed enhanced docetaxel bioavailability and tumour regression compared to Taxotrene®. NLCs loaded with Ibrutinib were developed using a Quality by Design approach for the treatment of chronic lymphocytic leukaemia. Nanoparticles of 106 nm and encapsulation efficiency of 70% demonstrated improved pharmacokinetic results with increased Cmax (2.9-fold), AUC (5.3-fold) and mean residence time (1.8-fold) in comparison to the free Ibrutinib. The clinical efficacy of NLCs was also better when lymphatic uptake was investigated by blocking chylomicron flow blocking by incorporating cycloheximide.

In the current study, we developed curcumin-loaded NLCs for the treatment of prostate cancer. The nanoparticles presented excellent physical stability under storage and showed a dose-dependent effect on cellular uptake and cytotoxicity. In vivo animal studies demonstrated significant tumour regression when compared to bulk curcumin.

## Materials

Tristearin, oleic acid, stearic acid, Tween 80 and curcumin were purchased from Merck (Gillingham, UK). Lutrol 407 was kindly provided by BASF (UK). All other solvents such as acetonitrile (ACN), ethanol (EtOH) and chemicals were of analytical grades. LNCaP cell lines were obtained from American Type Culture Collection (Virginia, USA). Thiazolyl blue tetrazolium bromide (MTT), Dulbecco’s modified Eagle’s medium (DMEM), Penicillin streptomycin, L-glutamin, heat inactivated fetal bovin serum (FBS) and trypsin were also acquired from Merck (UK). The Annexin V Apoptosis Detection Kit I was purchased from BD Biosciences.

## Methods

### Fabrication of NLC nanoparticles

The NLCs were prepared by means of a pre-emulsification step followed by high-pressure homogenization (HPH) with the lipid phase, comprising a blend of oleic and stearic acid. Briefly, NLC-containing suitable quantities of both solid and oily lipids, surfactants (Lutrol 407, Tween 80 with or without curcumin (CRN) are used, as shown in Table 1. The volume of the aqueous phase was kept at 50 ml. The NLCs comprised blank or CRN loaded which were primarily heated over the lipid’s melting point. For the CRN-loaded NLCs, the drug was dissolved in 3 ml of ethanol and added in the molten lipidic blend and subsequently dispersed in the surfactant solution (80 °C) and processed using an UltraTurrax T25 homogenizer (IKA® GmbH, Germany) to obtain the pre-emulsion. The crude dispersion obtained from the pre-emulsification step was placed in a Micro DeBee (South Easton, MA, USA) HPH and processed continuously for 7 min at 70 °C and 15,000 PSI. The final NLC dispersions were allowed to cool down at ambient temperature and the lipid to crystallize forming a solid matrix of lipid nanoparticles.

**Table 1:** Formulations of blank and drug-loaded NLCs

**Formulations**	**Tristearin (mg)**	**Oleic acid (mg)**	**Lutrol 407 (mg)**	**Tween 80 (mg)**	**CRN (mg)**	**Water (ml)**
**BL-NLCs**	400	250	150	100	90	50
**CRN-NLCs**	400	250	150	100	90	50

### Particle size and ζ-potential analysis

The obtained NLCs were evaluated for their particle size and ζ-potential using dynamic light scattering with the Zetasizer Nano-ZS (Malvern, UK). The nanoparticles were diluted (2–3 droplets) in purified water (dispersant), and each sample was measured in triplicate.

### Evaluation of drug loading and encapsulation efficiency

The encapsulated CRN in the NLC was estimated through a UV–vis spectrophotometer at λmax of 425 nm by constructing a calibration curve (*R*^2^ 0.999) of drug concentrations varying from 1 to 30 µg/ml (Douroumis 2011). Briefly, 1 ml (× 3) of nanoparticles were centrifuged for 30 min at 14,000 rpm and 25 °C. The collected pellets of CRN were dissolved in ACN to measure the CRN absorbance followed by suitable dilutions. The determined CRN amounts provided the accurate drug loading (DL) and the encapsulation efficiency (EE) using Eqs. 1 and 2:

1 $$DL=\frac{\text{Amount of CRN in NLCs}}{\text{Amount of NLCs}} \times 100$$

2 $$EE=\frac{\text{Drug loading}}{\text{Theoretical drug loading}} \times 100$$

### CRN release from NLCs

The CRN release from the nanoparticles was estimated by transferring 1 ml (× 3) of NLCs into dialysis bags (cellulose grade with 10,000 molecular weight cut-off), and placed in beakers comprising 0.5 l ddH_2_O and EtOH with 50:50, v/v ratio at 37 °C, similarly to Yang et al. [16]. All beakers were introduced in a water bath shaker (Thermo Scientific Precision TSGP15D) and at different time intervals, the dissolution media were collected and exchanged with additional 0.5 L of the same media. The CRN amounts were estimated spectrophotometrically as before (λmax 425 nm).

### Cytotoxicity studies

LNCaP cancer cells were cultured using Dulbecco’s modified Eagle’s medium with the addition of serum (10.0%), L-glutamine (1.0%) and penicillin streptomycin (1.0%) at 37 °C while 5% CO_2_ was supplied. The DMEM medium was replaced after 3 days.

The cytotoxicity of bulk CRN and CRN-NLCs was estimated using MTT assay with LNCaPs seeded in 24-well plates with cell densities of 1 × 10^6^ cells/well after 24 h incubation. Appropriate amounts of MTT solution (100 μl, 5 mg/ml) were added in each plate and incubated for another 2 h at 37 °C. Subsequently, the medium was rejected, and acidified isopropanol (200 μl) was added to solubilise the formazan crystals, which were placed into 96-well plates for absorbance measurements (492 nm) with a microplate reader. CRN amounts varied at concentrations from 10 to 100 µg/ml. Similarly, BL-NLC and CRN-NLC dispersions were incubated at 0.10, 0.20, 0.40, 0.60 and 0.90 mg/ml.

### Cellular uptake

The cellular uptake of NLC dispersions was determined after seeding 20 × 10^3^ cells/well on 24-well plates. The CRN-NLC samples were incubated with LNCaP cell for 24 h at different concentrations. After 24 h, the cell medium was removed and rinsed with a phosphate buffer solution (3 times). The cells were left in the dark for 15 min after the addition of 1 ml paraformaldehyde (4%) to fix them on the cover slips and subsequently mounted on glass slides with a vectashield medium enclosing DAPI. The uptake of CRC-NLCs was determined using the CRC fluorescence intensity which excites at 425 nm and emits at 530 nm [17]. Fluorescent images were collected with a Nikon ECLIPSE 90i microscope coupled to a Nikon (DS-Qi1Nc) digital camera using the NIS-Elements Advanced Research software. For the imaging studies, an oil immersion CFI Plan Apochromat VC 60 × N2 was used.

### Flow cytometry analysis

Due to CRN intrinsic green fluorescence, the cellular uptake was quantitatively estimated by Flow cytometry. As previously, 20 × 10^3^ cells/well were seeded on cover slips in 24-well plates, while the cells were incubated with CRN (ethanolic solution) and NLC dispersions (10 µg/ml) for 24 h. After discarding the medium and washing three times with PBS, the cells were separated from the well plates by adding EDTA. At the end of the trypsinization process cells were again rinsed three times and dispersed in 500 $\mu$ L of PBS for analysis.

### Studies of in vitro apoptosis

Briefly, LNCaP cells were seeded in 24-well plates and incubated for 48 h with cell densities of 1 × 10^6^ cells/well. Subsequently, the cells were centrifuged, collected, rinsed three times with PBS and re-suspended in 1 × binding buffer (500 mL), 7-amino-actinomycin (7AAD, 5 µl), PE Annexin V (5 µl) and incubated for 10 min under dark. The prepared samples were examined for the 7AAD and PE expression using the Accuri C6 flow cytometer with a blue laser (488 nm) and a suitable detector (FL1 path; 530/30 nm filter). A least 10 k of gated events was attained from each cell population, and the analysis was conducted with the Accuri software.

### Animal studies with mice bearing LNCaP xenografts

Animal studies were conducted using the protocol described by Yang et al. [16] with minor variations. For the purposes of the study, female nude mice aged 6–8 weeks were preserved in 12 h light/24 h dark cycle. LNCaP cells were cultured in DMEM, and the xenografts were developed after the injection of the cells (2 × 10^6^) in the fat pad of mammary glands. The tumours grew for 3 days (no treatment provision), and the volumes were monitored daily through recording two perpendicular tumour diameters with a calliper:

$$\lbrack\mathrm{tumour}\;\mathrm{volume}\;\lbrack\mathrm{mm}3\rbrack\:=\:(\mathrm{length}\;\lbrack\mathrm{mm}\rbrack)\:\times\:(\mathrm{width}\;\lbrack\mathrm{mm}\rbrack)^2\:\times\:0.52$$

The mice bearing LNCaP xenografts were allocated into groups (*n* = 6) in order to receive the required treatment: (1) blank control, (2) CRN ethanolic solution, (3) BL-NLC and (4) CRN-NLC. Each sample was administered through intravenous injection via mice tail with CRN treatment of 20 mg/kg. The injections were repeated twice per week at 200 µl per injection for 30 days. The tumour size and the mice weight were measured and recorded twice a week.

### Statistical analysis

The experimental findings are presented as means and standard deviation of the mean (*n* = 3). *T*-test analysis was introduced to investigate the differences between blank CRN and CRN-NLC dispersions while the effects were considered statistically significant only when the probability factor *p* < 0.05.

## Results and discussion

### NLC particle size and ζ-potential

**Fig. 1:**
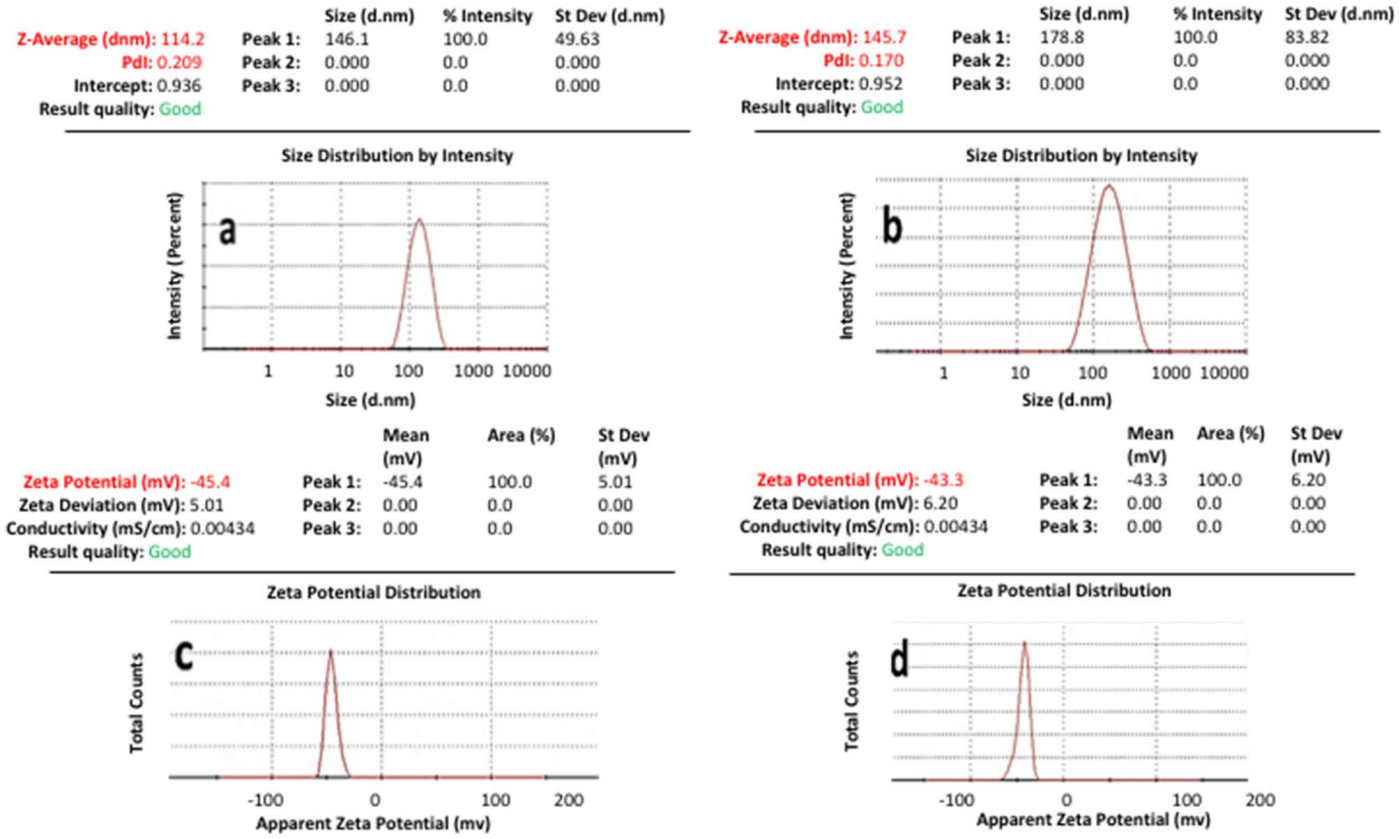
Particle size distribution and ζ-potential of **A**, **C** BL-NLC, **B**, **D** CRN-NLC respectively

The obtained BL-NLC and CRN-NLCs were successfully prepared using HPH by adjusting the homogenization temperature and the applied pressure. All materials used for the NLCs including the lipids, surfactant and oily phase were generally recognised as safe (GRAS). The freshly made BL-NLC and CRN-NLC are characterised in terms of both particle size distribution and ζ-potential, as shown in Table 2 but also evaluated after 6 months storage stability at 4 °C. Figure 1 shows that the particle size of blank and CRN-loaded NLCs varied from 110 to 150 nm. The drug encapsulation led to a slight particle increase of about 35 nm. The produced nano dispersions presented a monomodal particle size distribution for blank CRN-loaded formulations.

**Table 2:** Particle size and zeta potential of NLC formulations (*n* = 3)

	**Months**	**Particle size (nm)**	**Zeta potential (mV)**
**Blank NLC**	1	113.7 ± 1.2	− 44.1 ± 1.4
	3	115.0 ± 0.8	− 44.1 ± 0.3
	6	118.3 ± 1.3	− 42.9 ± 0.9
**CRN-NLC**	1	146.5 ± 1.1	− 42.8 ± 0.6
	3	147.7 ± 1.7	− 41.1 ± 1.0
	6	149.6 ± 2.4	− 40.6 ± 0.7

HPH was optimized (data not shown) to produce NLCs smaller than < 150 nm in order to assure their acceptability for cancer treatment as size plays a critical role in cellular uptake monomodal particle size distribution, while particle decrease results in improved bioavailability [18].

Furthermore, the observed polydispersity indexes for all NLCs were around 0.2 indicating uniform size populations. The measured ζ-potential showed negative surface charge and varied from 40 to 45 mV. Those values were considered indicative of NLC long-term stability as colloidal nanoparticles are considered stable when ζ-values are greater than 30 mVas a result of the electric repulsion [19]. For the CRN-NLCs (− 42.8 ± 0.6) a small ζ-potential decrease was observed compared to BL-NLC (− 44.1 ± 1.4) after 6 months storage stability. The reduction can be explained through the drug partial absorption on the NLC surface resulting in masking the surfactant negative charge [19].

### Evaluation of the DL and EE

Further investigation of the CRN-NLC dispersions showed good DL and EE with 9.3% and 92.9%, respectively. Moreover, the high EE of the CRN-loaded nanoparticles denoted the efficiency of HPH for the encapsulation of lipophilic compounds with negligible drug losses during homogenization. The achieved DL and EE of CRC-NLCs were related to the lipophilic nature of the drug and the less ordered NLC crystal lattice which favours the incorporation of drugs in the lipidic matrix [20]. The DL and EE findings were in good agreement with previous studies that demonstrated similar outcomes [21].

### Drug release studies

**Fig. 2:**
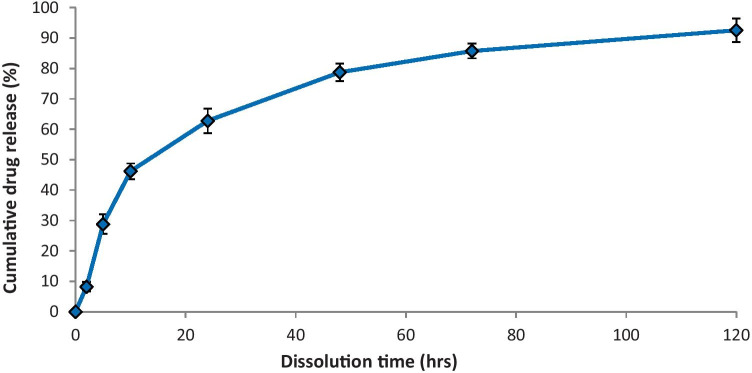
In vitro CRN release from NLCs

The practicality of employing NLC to distribute CRN was one of the main topics explored in this study. As illustrated in Fig. 2, the potential of NLC to deliver CRN is investigated by evaluating the drug release. The release test lasted 120 h at a constant temperature of 37 °C. Because of the hydrophobic nature of CRN, the release tests were carried out in 50% v/v ethanol solutions, with CRN solubility of 0.693 ± 0.13 mg/ml, as proposed by Kakkar et al. [22].

Two distinct trends appear to characterise the CRN release pattern. Over the first 10 h, there was a burst effect, followed by a steady drug release from NLCs for the remainder of the monitoring period. After 48 h, the cumulative release rate was 78.8% where over the next 72 h, this figure increased to 92.6%. The discovery is in line with other researchers’ results on CRN release from lipid nanoparticles [20]. Despite the fact that the components utilised to make CRN-loaded lipid nanoparticles differed from those used in this study, the obtained results can be justified: The absorbed CRN on the surface of NLC causes the burst release, while the time necessary for the drug to diffuse into the dissolution medium from the NLC causes the sustained release [23].

### CRN antiproliferative effect

The tumour killing activity of CRN-loaded NLC formulations was assessed using the MTT test against LNCaP prostate cancer cells to measure their cytotoxicity. The antiproliferative activity of CRN-loaded NLC formulations was tested to see if CRN’s efficacy was maintained after encapsulation in a lipid matrix. The antiproliferative activity of pure CRN on the LNCaP cell lines was investigated initially, and the CRN-NLC drug delivery system was compared.

**Fig. 3:**
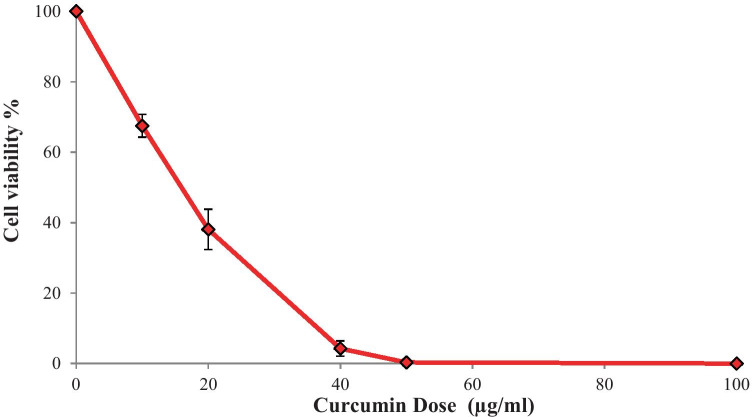
MTT assay used to test the antiproliferative effects of pure CRN on LNCaP cells for 24 h. (*n* = 3)

As illustrated in Fig. 3 after 24 h of incubation, bulk CRN showed excellent antiproliferative efficacy. CRN presents some of the most sought properties of a cancer therapeutic drug, where preferential killing or therapeutic selectivity is one of them. Hence, CRN can be used to selectively target cancer cells while causing minimal harm to healthy cells [24]. The in vitro antiproliferative effect of CRN was substantial, indicating CRN’s efficacy against LNCaP cancer cells.

### NLC’s antiproliferative effect

The cytotoxicity of blank NLCs is a crucial consideration when using it as a drug career, while lipid nanoparticles with low cytotoxicity are required for cancer applications. To assess the effect of blank NLCs, their cytotoxicity was tested on LNCaP prostate cancer cells.

**Fig. 4:**
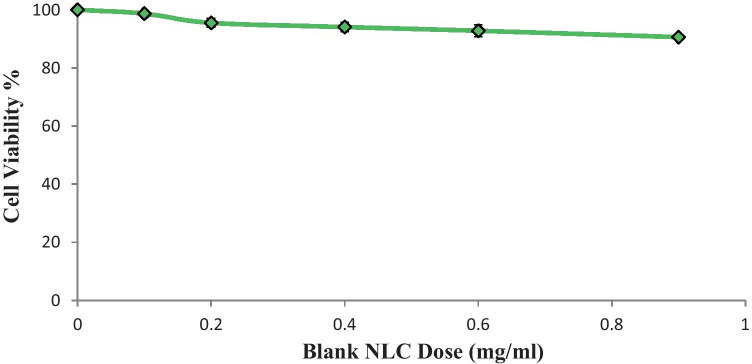
Cell viability of blank NLCs in LNCaP cancer cells. (*n* = 3)

Blank NLCs had no influence on the cytotoxicity of the LNCaP cancer cells as shown in Fig. 4. After 24 h of incubation at a high NLC concentration (0.9 mg/ml), cell viability was 91.0% which is deemed insignificant [25].

**Fig. 5:**
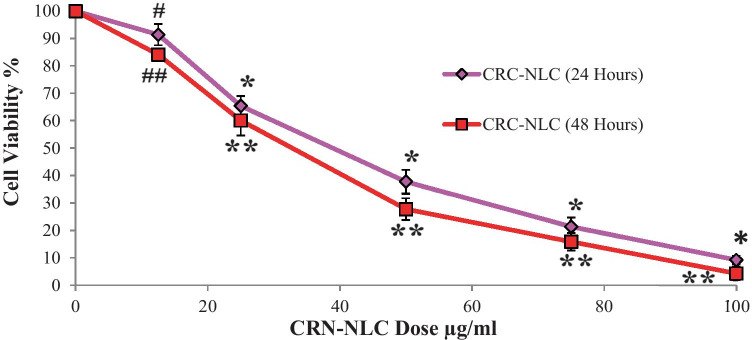
Cell viability of CRN-NLCs after 24 and 48 h ^#^*p* = 0.0323, *p < 0.0001, CRN-NLC (24 h) vs. BL-NLC and ^##^*p* = 0.0003, **p < 0.0001, CRN-NLC (48 h) vs. BL-NLC (*n* = 3)

The MTT assay was used to assess the antiproliferative impact of CRN-NLC. As demonstrated in Fig. 5, increases in CRN concentration had an influence on the viability of LNCaP cells, which was reduced to 91.5% at the lowest dose of 12.5 g/ml CRN concentration.

The antiproliferative action of CRN-NLC was even more pronounced at higher CRN concentrations of 50 g/ml and 75 g/ml, with cell viability reduced to 37.8% and 21.4%, respectively. The cell viability was further reduced to 9.2% at a dose of 100 g/ml, which is regarded very significant when compared to the blank NLC nanoparticles (*p* < 0.0001).

These results clearly show that CRN-NLCs have a dose-dependent antiproliferative effect on the LNCaP cells. However, as a continuous release pattern of CRN was observed in CRN-NLC release investigations, a further 48-h study on LNCaP cells was also undertaken to further investigate this finding.

The antiproliferative impact was considerably more pronounced after 48 h of CRN incubation, as expected. Cell viability was nearly 0% after extending the incubation period at 100 g/ml CRN concentration. Our experimental findings were similar to those reported by Fang in a previous study [21].

This CRN-NLC’s time-dependent cytotoxic effect is a clear evidence of CRN’s sustained release, as well as CRN-extended NLC’s inhibitory effect. Because blank NLCs presented no effect on the cytotoxicity of LNCaP cells, it was concluded that the CRN molecules were primarily the cause of the reduced cell cytotoxicity.

### Investigation of cellular uptake

CRN’s inherent fluorescence, which can be used directly to quantify its cellular uptake, is one of its advantages. The cellular uptake of CRN-NLCs was studied using fluorescence microscopy; laser intensity, offset, sensitivity, and gain constant were harmonised during cell imaging to better understand the internalisation of the CRN-NLC inside the cell.

NLCs are known to enter cells via endocytotic routes, according to Mohanty and Sahoo (2010). Their internalisation was confirmed by incubating 10–30 μM of native CRC and equivalent amounts in nanoparticulate formulations in PANC-1 cells for 24 h and qualitative cellular absorption experiments were carried out after incubation [26].

**Fig. 6:**
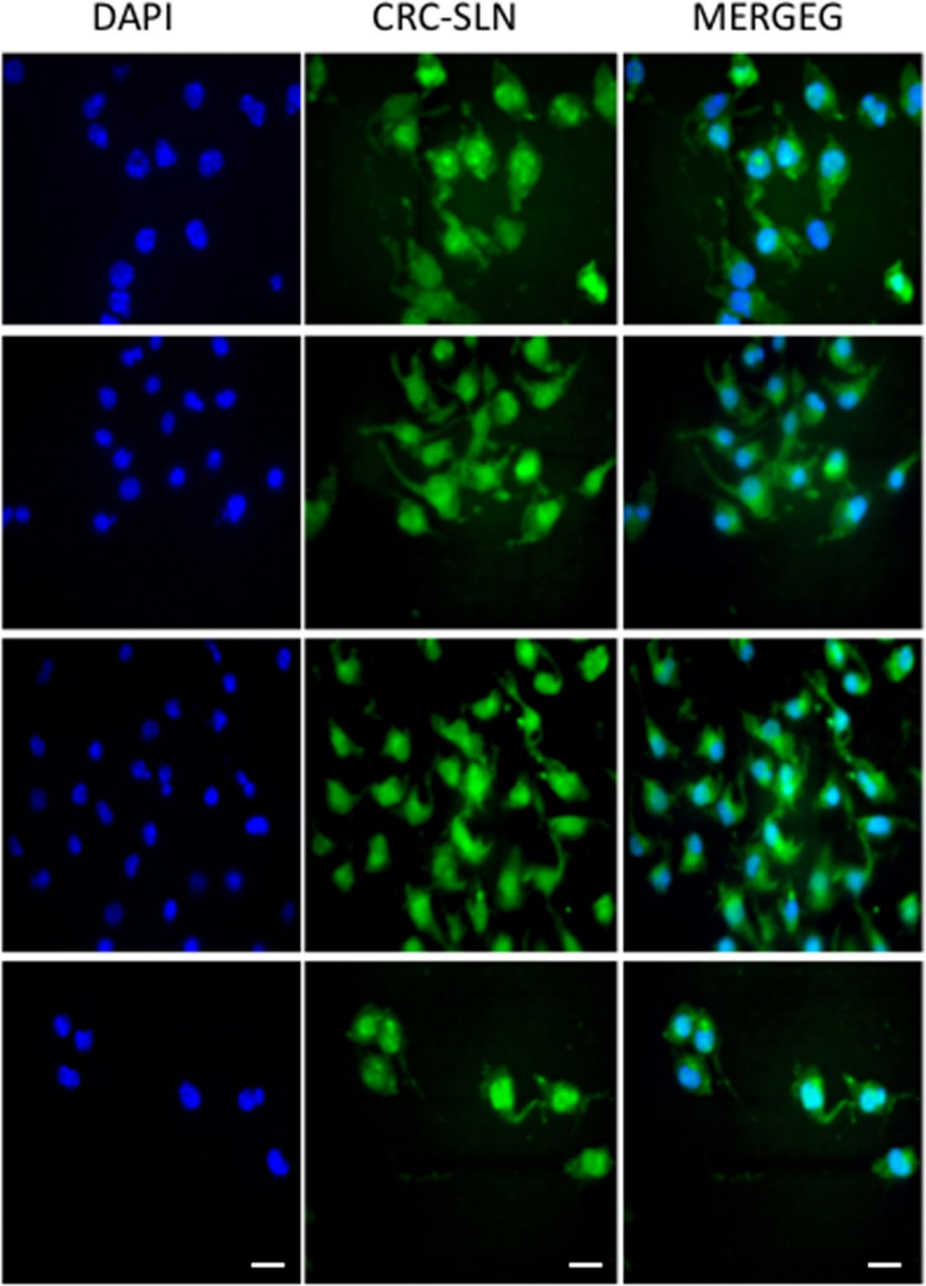
Cellular uptake of drug-loaded nanoparticle where the green colour shows internalization of CRC-NLCs. The nucleus was stained with the blue colour of (DAPI)

Prior to the experiment, the nucleus of the cell was labelled with DAPI, a DNA specific fluorescent probe [27], and the images were acquired after fixation. Figure 6 shows overlay pictures that clearly demonstrate green (CRN-NLC) and blue (nucleus) patches, showing CRN-NLC internalisation within the cytoplasm of the nucleus.

### Flow cytometry of NLCs

**Fig. 7:**
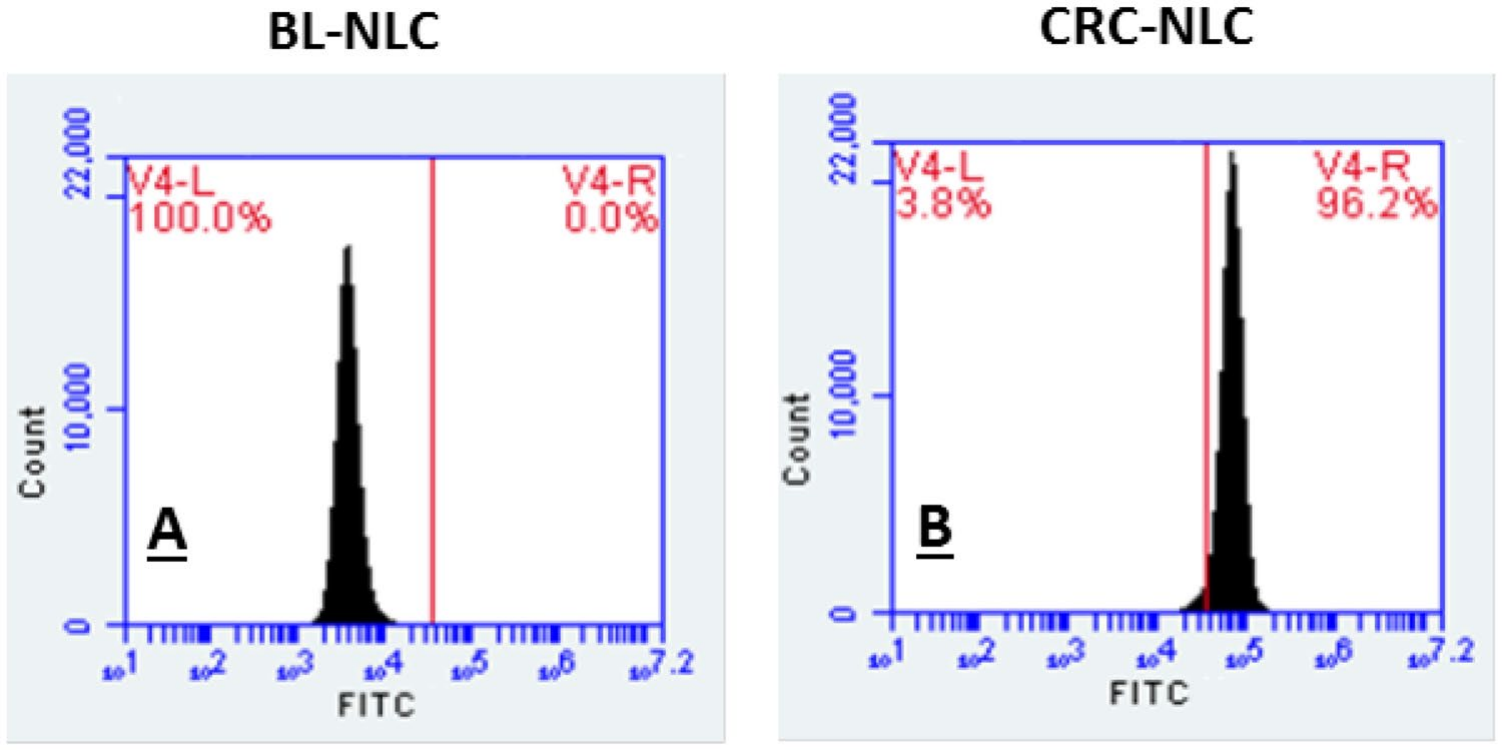
Cellular uptake of **A** BL-NLCs and **B** CRN-NLC by flow cytometric analysis

CRN accumulation on LNCaP cells after 24 h incubation of CRN-NLC is seen in flow cytometric histograms in Fig. 7. For each cell, the signal intensity was measured quantitatively, and the expression of a high proportion of cellular uptake is linked to high fluorescence intensity.

As shown in Fig. 7, CRN-NLC presents signal intensity of 96.2%, according to the histogram. Blank NLCs were also examined for any autofluorescence effects and interference with the fluorescent intensity produced by CRN-NLC. Figure 7 shows that no fluorescence intensity was detected, while the results from the quantitative cellular uptake of CRN-NLC matched the images captured by fluorescence microscopy.

### In vitro apoptosis

Apoptosis is a physiologically regulated cell death that happens during the growth of cells, and it is distinguished from necrosis. PS is exposed to the exterior cellular surroundings when phosphadylcholine (PS; located on the cytoplasmic side of a normal cell bilayer) is translocated from the inner to the outer leaflet of the plasma membrane during apoptosis [28].

PE Annexin V and 7AAD were used to stain both untreated and treated cells. Because Annexin V has a high affinity for PS, it can detect apoptosis at an early stage (Annexin V positive, 7AAD negative). The combination of 7AAD with PE Annexin V allows for the detection of early apoptotic cells. Dead and impaired cells are permeable to 7AAD, but live cells with intact membranes exclude it.

**Fig. 8:**
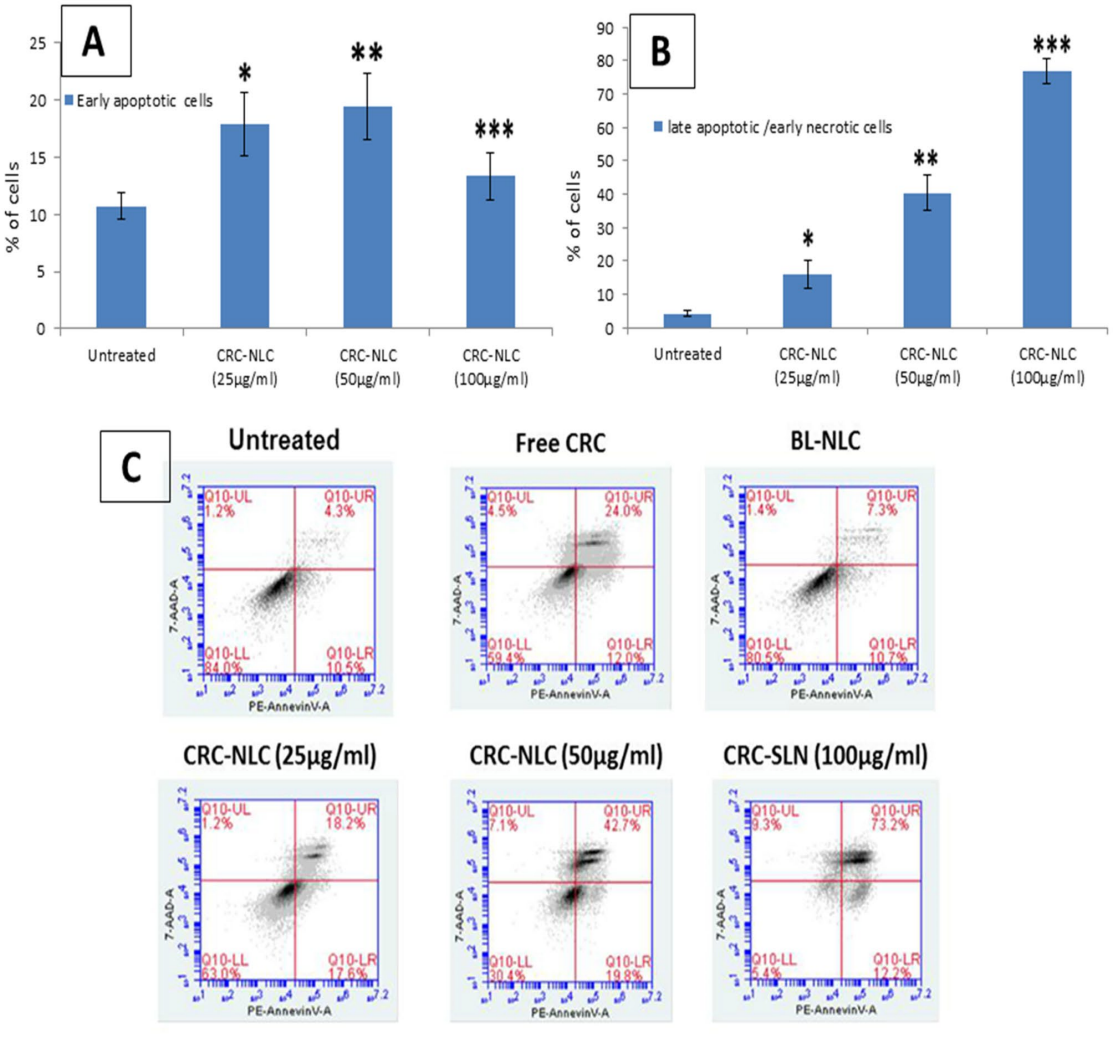
Quantitative apoptotic analysis in LNCaP cells treated with BL-NLC, pure CRN and CRN-NLC. **A** Treatment with a concentration of 25, 50 or 100 g/ml of CRN-NLC dosage for 24 h had a dose-dependent effect on early apoptosis, as evaluated by flow cytometry. **B** Treatment with concentrations of 25, 50 and 100 g/ml of CRN-NLC dosages for 24 h had a dose-dependent influence on late apoptosis, as measured by flow cytometry (bar charts). (*) *p* < 0.05, control vs. CRN-NLC (25 µg/ml), (**) *p* < 0.05, control vs. CRN-NLC (50 µg/ml), (***) *p* < 0.05, control vs. CRN-NLC (100 µg/ml) and **C** PE Annexin V vs. 7-AAD dot plots are used to represent dose-dependent effects. Top right: late apoptotic cells/early necrotic cells; Top left: necrotic cells; bottom right: early apoptotic cells; bottom left: live cells; and (*n* = 3)

Flow cytometry is used to identify and quantify the induction of apoptosis by CRN following the treatment on cells, as shown in Fig. 8. Pure CRN (15 g/ml) demonstrated 12% early apoptotic cells and 24% late apoptotic/early necrotic cells, while cells are treated with CRN-NLC nanoparticles. Cells treated with CRN-NLC (25 μg/ml CRN), revealed 17.6% and 18.2% early apoptotic (Annexin V + 7AAD) and late apoptotic/early necrotic (Annexin V + 7AAD +) populations, respectively. This showed that, like pure CRN, CRN-NLCs trigger apoptotic pathways. A considerable upsurge in apoptotic cells was seen at higher concentrations of 50 g/ml and 100 g/ml. In cells treated with CRN-NLC at 50 g/ml CRN dosage, the percentages of early apoptotic (Annexin V + 7AAD) and late apoptotic/early necrotic (PE Annexin V + 7AAD +) populations were 19.8% and 42.7%, respectively. Experimentation with untreated and BL-NLC controls revealed no apoptotic impact, implying that the apoptosis generated by the CRN-NLCs was due to encapsulated CRN.

### In vivo anticancer activity of CRN and CRN-NLC on LNCaP tumours

The blank NLC, pure CRN as well as CRN-NLC, were injected in mice xenografts of LNCaP tumours at doses of 20 mg/kg to investigate their in vivo anticancer activity. The prostate cancer cells were inoculated with a cell density of 1 × 10^6^ in mice, and the cells were allowed to develop for a total of 6 days before starting treatments with various formulations.

**Fig. 9:**
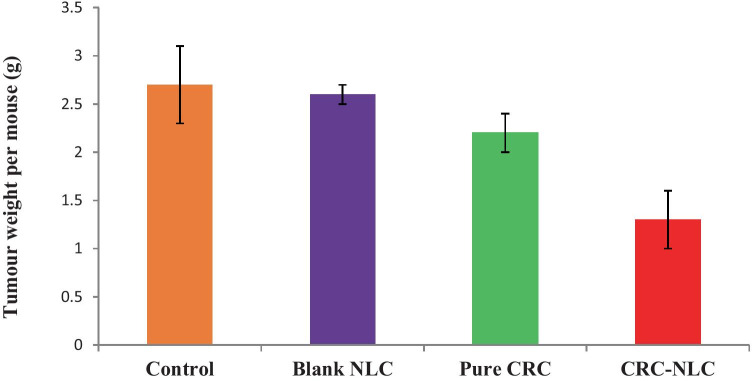
Comparison of tumour suppression on LNCaP prostate cancer of control, blank NLC, pure CRN, CRN-NLCs

Unpaired *t*-test was utilised to assess statistically significant differences between each set of NLCs, revealing the anticancer efficacy differences between each formulation. The anticancer activity of CRN is shown in Fig. 9 in comparison to the control and blank NLCs, with the data expressed in terms of mean tumour tissue weight in each group.

When CRN was administrated to mice with LNCaP tumours, the tumour size was reduced by 19% when compared to the control treatment. Furthermore, an unpaired *t*-test between these two groups revealed a significant difference, with *p* = 0.020. When mice with tumours were given CRN-NLCs, the anticancer impact was significantly stronger.

When compared to the control and the BL-NLC group, a tumour size reduction of 52% and 50% was seen after 4 weeks of therapy with CRN-NLC nanoparticles. The therapeutic efficacy of the CRN-NLCs was substantially higher when compared to the control and blank NLC groups, according to an unpaired t-test (*p* < 0.0001). The comparison of tumour weight between the bulk CRN and that of CRN-NLC, demonstrated that animal weight was reduced by 40% for the latter. This evidently demonstrates the benefit of the CRN usage as a drug delivery system for cancer treatment.

**Fig. 10:**
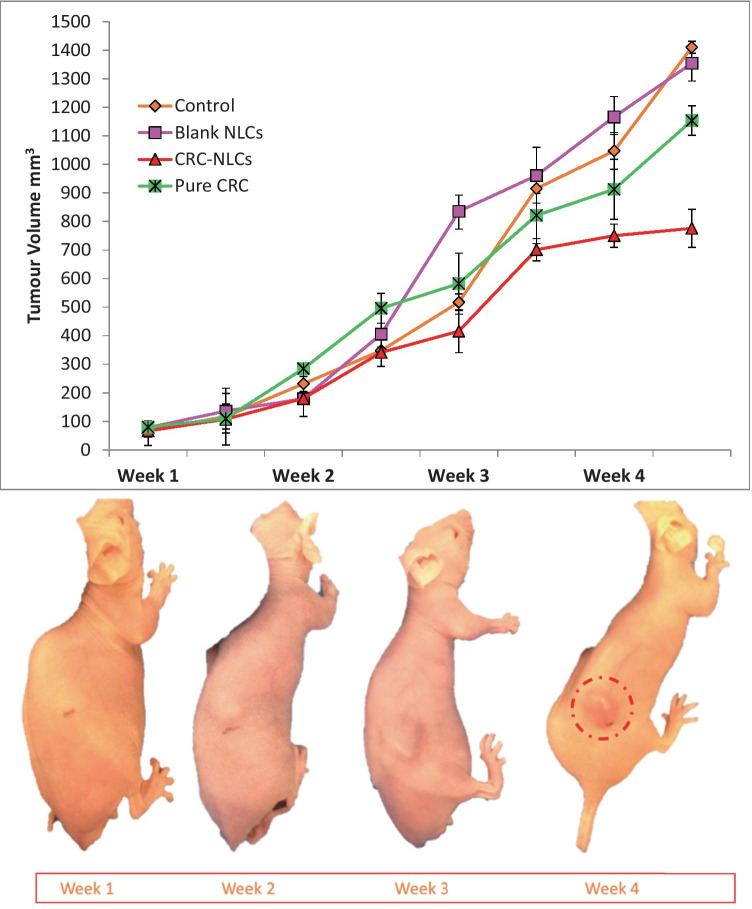
Therapeutic impact of control, blank NLC, pure CRN and CRN-NLC on tumour-bearing animals (top) and tumour growth in mice bearing LNCaP tumours after treatment with CRC-NLC

As the animal trials of pure CRN and CRN-NLC nanoparticles revealed anticancer action, they were measured in terms of tumour volume. As illustrated in Fig. 9, when compared to control and blank NLC-administrated mice, treatment with bulk CRN and CRN-NLCs resulted in considerable tumour regression of LNCaP xenografts. Figure 10 shows the findings of tumour volume changes as a function of time with CRN-loaded NLC. After 4 weeks of therapy, the average tumour variations in LNCaP tumours were 1412.0 mm^3^ (control), 1375.2 mm^3^ (blank NLCs), 1155.1 mm^3^ (pure CRN) and 778.4 mm^3^ (CRN-NLCs). When compared to control and BL-NLC, the NLC-CRN exhibited a substantial growth inhibitory activity (*p* < 0.0001). Furthermore, as compared to pure CRN (*p* < 0.0001), CRN–NLC demonstrated better anticancer activity. Another noteworthy finding was the tumour-suppressing properties of the BL-NLC nanoparticles. Figure 10 shows that, alike to control therapy (*p* = 0.1300), blank NLC did not demonstrate any substantial reduction in tumour suppression.

The weight of the mice was also monitored for 4 weeks during the dose administration, and no weight changes were seen in any of the animals that received the treatment. Figure 2S (supplementary data) revealed that the nanoparticles were well tolerated by the mice, who showed no evidence of toxicity or considerable weight loss.

Despite the fact that CRN is known to be highly effective in commencing protection against cancer in animals exposed to a range of chemical carcinogens, CRN’s bioavailability is known to be low [29]. CRN can also decrease cell proliferation and signal transduction activation in cancer cells that present either androgenic or non-androgenic activities. CRN has been reported to decrease both constitutive and inducible nuclear factor-B and so has high antioxidant and anti-inflammatory properties [30].

CRN encapsulation in NLC can help to alleviate some of the bioavailability issues associated with intravenous delivery of CRN, allowing for optimal anticancer activity when delivered at the tumour site. According to previous studies, drug encapsulation can lead to increased CRN plasma concentrations, which could be due to the nanoparticle’s small size; this, in turn, facilitates the NLC circulation for prolonged time which may result in a higher anticancer efficacy [31, 32]. Similar experimental outcomes were found by Chen et al. (2012) where CRN and CRN-NLC administration successfully slowed tumour growth and prolonged mice survival but did not complete eliminate tumour growth [33].

## Conclusions

In the current work, CRN-NLC nanoparticles were effectively produced by employing high pressure homogenization with narrow particle size distribution, low ζ-potential (− 40 mV) values, and long-term stability. CRN was encapsulated efficiently within SLNs, and in vitro release tests revealed prolonged release. Cytotoxicity experiments demonstrated considerable cell growth suppression after treatment with CRN-NLC, and cell viability reduced to 4.3% at a CRN dose of 100 μg/ml. Flow cytometry analysis demonstrated CRN-NLC apoptosis, with 76.9% of cells on late apoptotic/early necrotic cells. In vivo investigations on LNCaP cancer xenografts demonstrated that CRN-NLC nanoparticles had a much higher anticancer efficacy than empty NLCs and pure CRN. CRN-NLC nanoparticles showed considerable tumour suppression, rendering it a suitable drug delivery system for the treatment of prostate cancer.

## Supplementary Information

Below is the link to the electronic supplementary material.

Supplementary file1 (DOCX 27 KB)

## Data Availability

N/A.
