# Supplementary material for: In vivo evaluation of nanostructured lipid carrier systems (NLCs) in mice bearing prostate cancer tumours
Source: Drug Deliv Transl Res. 2021 Nov 29;13(8):2083–95. doi: 10.1007/s13346-021-01095-1 (PMC10315352; doi:10.1007/s13346-021-01095-1)
Supplement: Supplementary file 1 — Supplementary file1 (DOCX 27 KB) [file 13346_2021_1095_MOESM1_ESM.docx]

**Supplementary data**

**Fig. 1:** Effect in therapeutic efficacy of control, pure CRN and CRN-NLC nanoparticles

on tumour bearing animals.

**Fig. 2:** Administration tolerance of tumour-bearing mice over a four-week treatment period
